# How dairy heifers initially respond to freestalls: The effect of neck-rail placement

**DOI:** 10.3168/jdsc.2024-0601

**Published:** 2024-07-26

**Authors:** Marek Gaworski, Kathryn McLellan, Marina A.G. von Keyserlingk, Daniel M. Weary

**Affiliations:** 1 Animal Welfare Program, Faculty of Land and Food Systems, The University of British Columbia, Vancouver, Canada V6T 1Z6; 2 Institute of Mechanical Engineering, Warsaw University of Life Sciences–SGGW, 02-787 Warsaw, Poland

## Abstract

Dairy heifers are often kept on open packs early in life and later transitioned to freestalls. The neck rail in freestalls acts as a barrier intended to prevent animals from entering too far in the stall, with the aim of improving stall cleanliness. Unfortunately, this barrier can hinder stall use and may be a hazard to animals due to physical contact, perhaps especially when animals first learn to use the stall. The primary aim of this study was to investigate the effects of 2 different neck-rail positions on frequency of stall use (for lying and standing) and misuse (standing or lying backward in the stall and lying down outside of the stall) by dairy heifers when first introduced to freestall housing. A secondary aim was to measure, via the use of an instrumented neck rail, the maximum force (N) with which heifers contacted this part of the stall. Holstein heifers (n = 8 per treatment) were randomly assigned to one of 2 neck-rail positions: 110 and 130 cm (as measured diagonally from the inside of the rear curb to the bottom of the instrumented neck rail, 0.7 m above the bedded stall surface). Animals were tested individually and responses were measured for 6 h following introduction to the freestalls. On average (median, minimum, maximum), heifers lay down 3.5 (0, 6) times in the freestall when the neck rail was positioned at 130 cm versus 0 (0, 4) times at 110 cm. Heifers also were more likely to stand fully in the stall when the neck rail was positioned at 130 cm versus 110 cm (3.5, 0, 9 vs. 1, 0, 3), and were also more likely to stand backward in the stall (3, 0, 5 vs. 0, 0, 1). In contrast, heifers tested at the 130 cm position were rarely observed lying down outside of the stall (1, 0, 4), whereas this behavior was more common at the 110 cm treatment (4, 0, 7). Contrary to our expectation, the force with which heifers contacted the neck rail was higher in the 130 cm versus 110 cm treatment (337, 6, 548 vs. 78, 26, 403 N). We conclude that neck-rail position acts as a barrier for naïve heifers, such that a more restrictive position reduces the likelihood that heifers use the stall correctly.

## Highlights

• Naïve heifers were observed when first introduced into an instrumented freestall.
• Neck rails in the stall were assessed at 2 positions: 110 or 130 cm from the curb.
• The 110 cm position reduced lying in the stall and increased lying in the alley.
• The 130 cm position increased the maximum force of contact with the neck rail.

## Graphical Abstract

**Figure:**
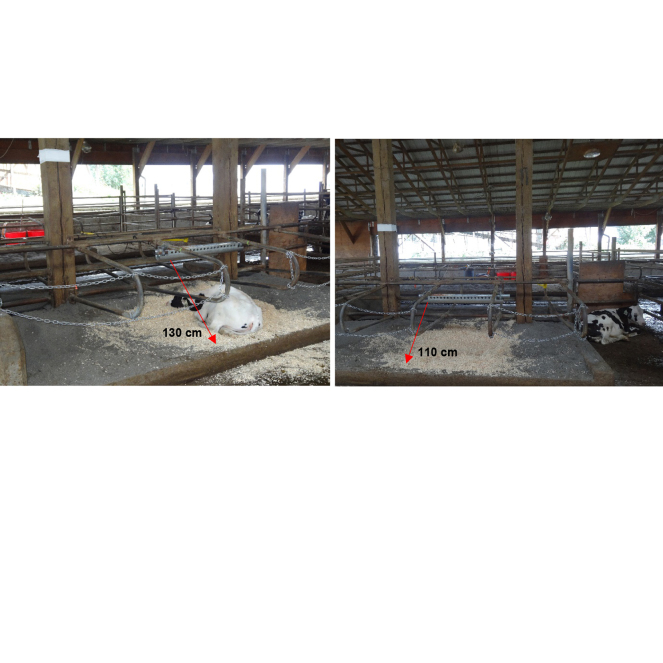
**Summary:** We tested the effect of 2 different neck-rail positions on stall use (for lying and standing) and misuse (standing backward in the stall and lying down outside of the stall) by dairy heifers when first introduced to freestall housing. The neck rail was also equipped with sensors to measure the maximum force of any contact from heifers as they lay down or stood up in the stall. We found that heifers were less likely to use stalls for lying and standing when the rail was positioned more restrictively, and were more likely to spend time lying down in the alley outside of the stall. Heifers contacted the neck rail with considerable force, and this force was higher in the stalls with less restrictive neck rails.

Neck rails should be positioned such that the stall is comfortable for the cow while standing and lying but also encourages positioning such that, if she defecates or urinates while standing in the stall, the urine and feces go into the alley rather than onto the stall surface. Positioning a neck rail more restrictively improves stall and udder cleanliness (Fregonesi et al., 2009), but also increases the time spent standing with 2 hooves in the stall (Tucker et al., 2005), increasing risks of lameness and hoof-related diseases (Bernardi et al., 2009). Epidemiological studies confirm the importance of neck-rail placement. For example, one study concluded that correct positioning of the neck-rail was important for preventing injuries (Veissier et al., 2004), and another concluded that neck-rail placement was a risk factor for dirty dairy cows (Ruud et al., 2010). These studies focused on cows already habituated to stalls; little work has investigated neck-rail positioning when cattle are first introduced to freestalls.

When introduced to freestall housing cattle may refuse to enter and instead lie down in the alley (O'Connell et al., 1993; Kjæstad and Myren, 2001) or misuse the stall (e.g., by lying backward in the stall; von Keyserlingk et al., 2011; Van Os et al., 2021). Previous work has indicated that the presence of a neck rail may affect stall use; heifers introduced into a freestall without a neck rail spent less time standing with 2 hooves in the stall as compared with when a neck rail was present (von Keyserlingk et al., 2011; testing cows over a 5-d period). To our knowledge no work has examined the effect of neck-rail position on how naïve cattle use freestalls.

Positioning a neck rail further from the rear curb may reduce the risk or force of contact and also reduce the chances of injury (Zaffino Heyerhoff et al., 2014), particularly for larger animals most affected by restrictive neck-rail positions (Tucker et al., 2005). To our knowledge, only one previous study (Blom et al., 1984) measured the pressure applied to the neck rail by cows, but no work has assessed the effect of neck-rail position on this measure.

The objectives of this study were to investigate the effects of 2 neck-rail positions on (1) freestall use and misuse, and (2) the force exerted on the neck rail, when naïve dairy heifers were introduced into freestalls. We predicted that positioning the neck rail further from the curb of the freestall would increase the number of times heifers lay down, stood fully in the stall and stood backward in the stall, and would reduce the number of times they lay down outside of the stall and the force with which they contacted the neck rail.

This study took place at The University of British Columbia Dairy Education and Research Centre (BC, Canada) in August 2019. All procedures were approved by The University of British Columbia Animal Ethics committee under protocol #A15–0082.

Sixteen Holstein heifers (mean ± SD), 102.6 ± 5.2 d of age and BW of 127.8 ± 11.32 kg (range 101.5 to 146.0 kg), were used in the study. Heifers averaged a wither height of 99.8 ± 3.0 cm (range 93.0 to 104.0 cm; vertical distance from ground level to the highest point of withers), hip height of 104.9 ± 3.0 cm (range 100.0 to 110.0 cm; vertical distance from ground level to highest point of hook region) and body length of 83.7 ± 2.7 cm (range 77.0 to 88.0 cm; horizontal distance from first cervical vertebra to most caudal vertebra at tail). No a priori power analysis was conducted. Earlier work on heifer responses when introduced to freestalls (von Keyserlingk et al., 2011; testing 7 groups in one experiment and 12 in another); thus, we considered more replicates (n = 16) but fewer heifers, as these were tested individually.

Heifers were housed in a naturally ventilated north-south oriented wood-frame barn (42 × 36 m). Average daily temperature (HOBO Prov v2 Temp/Rh logger, Onset Computer Corporation, Bourne, MA) was 20.6 ± 1.8°C. Heifers were group housed in 2 home pens (11.7 × 4.9 m) in groups of 9 ± 2 animal. These 2 pens were faced one another, separated by a drive-through feed alley. Group composition was dynamic, with new heifers added and removed throughout the duration of the study. Each home pen contained an open sawdust bedded-pack area (8.6 × 5.0 m, average bedding depth of 18 cm), a concrete feed alley (3.1 × 4.9 m), and a concrete drinking area (2.0 × 1.3 m). A diagonal slanted feed barrier with 14 feeding spaces was positioned along the feed alley with barriers located every 24 cm (25 cm center to center). The feeding areas was scraped 6 times daily with automatic manure scrapers and new sawdust was added once weekly into the bedded-pack.

We used an experimental pen with 4 freestalls, configured in one row (Figure 1). This pen was on the same side of the barn as one of the home pens, but separated by one other pen (also 8.6 m wide, housing older, familiar heifers), thus allowing visual and vocal contact with pen mates in the home pen, and nose-to-nose contact with the heifers in the adjacent pen. The stalls measured 1.8 m in length and were separated using stall partitions spaced 0.9 m center to center. The length of the lunge space was 0.5 m. Three of the 4 stalls were blocked off with chains such that one stall was available for use. The stall base was sand; on top of this sand surface, we added approximately 15 cm of sawdust so that bedding was level with the rear curb. The instrumented neck rail (with metal push-pipe: dimensions 1.2 × 0.1 × 0.1 m, rigidly connected to the neck rail; designed by MG and manufactured by ZEPWN, Poland) was attached to stall partitions such that the bottom of the rail was 0.7 m from the surface of the bedding. The device measured the force (N) applied by the heifer using sensors (Utilcell M350a, Spain; measuring forces up to 3,000 N with an accuracy of 0.3 N recording at 10 Hz). Every day before testing the instrumented neck rail was calibrated using an auto-calibration function.

**Figure 1:**
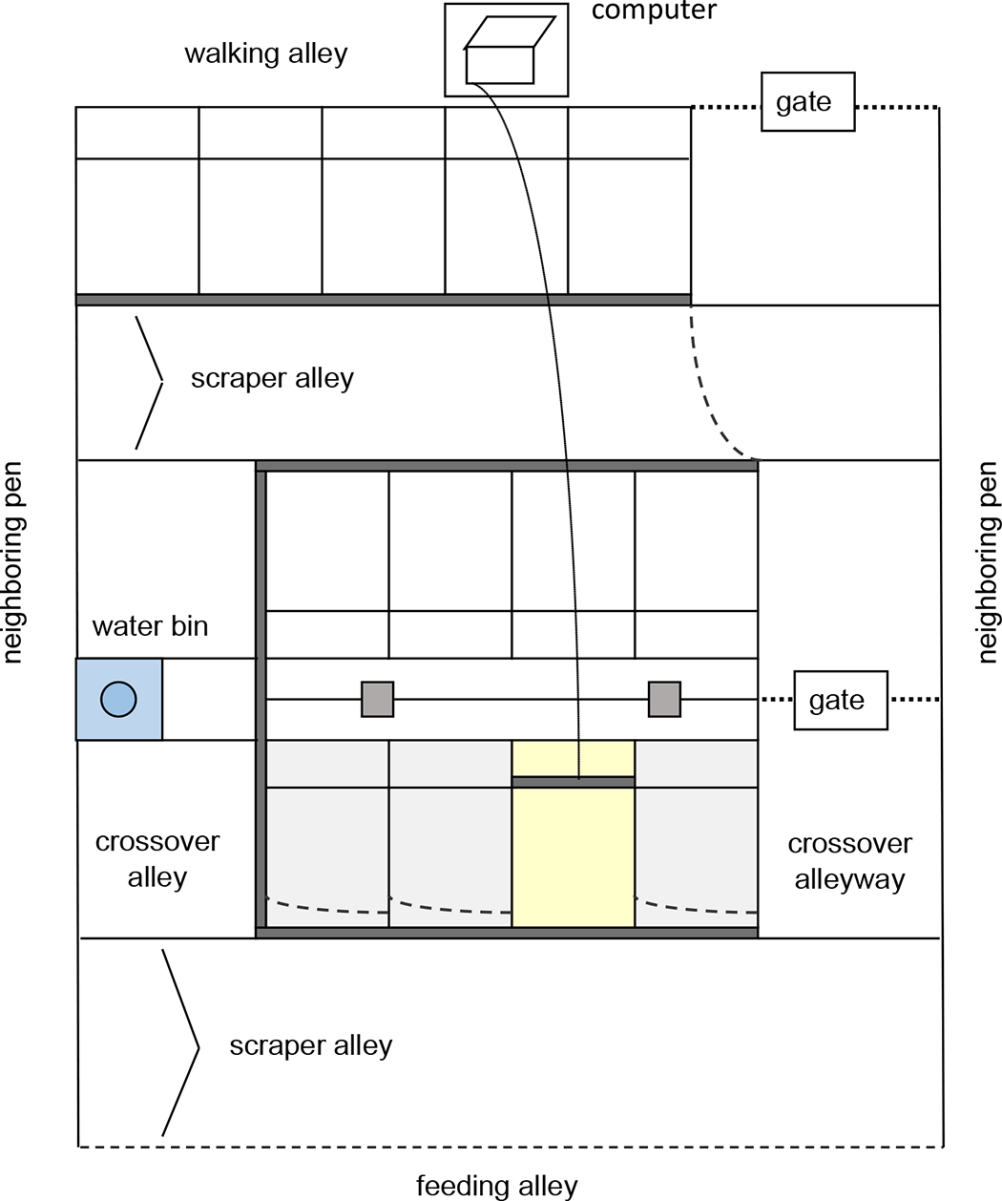
Layout of the experimental area. Only 1 stall was accessible (shown in pale yellow); this stall was equipped with an instrumented neck rail able to measure force exerted by the heifer. The instrumented neck rail was 120 cm wide (i.e., wider than the width of the lying stall); the length of the apparatus had no effect on the force measured as sensors were designed such that regardless of where the force was applied, the device showed the same value. In the pen, heifers had access to the marked areas (crossover alley, scraper alley, and the lying stall; heifers could feed from the feeding alley).

The stall was cleaned daily and fresh sawdust added to maintain a constant bedding depth. The concrete alleyway (7.0 × 3.1 m) located between the freestalls and the feed bunk was scraped 6 times daily with automatic manure scrapers. Two concrete crossover alleyways (1.2 × 2.0 m) were located on either side of freestalls, perpendicular to the feed bunk. The feed bunk was equipped with head-lock feed barriers (7.0 m) containing 13 feeding places, spaced 48.5 cm center to center. All heifers were group housed in the home pen for 14 ± 4.8 d before the start of the experiment. No heifers had previous experience in a freestall before the study. All pens were provided ad libitum access to fresh feed (grass hay and a concentrate). Home pens were fed daily at 0900 h, with feed pushed up at 1100 and 1600 h. Feed delivery was at 1200 h for the experimental pen. Fresh water was available in all pens via self-filling troughs.

Heifers were assigned to one of 2 neck-rail positions: 110 or 130 cm from the rear curb, measured diagonally from the inside of the rear curb to the bottom of the instrumented neck rail. Heifers were tested in order of birth day, alternating treatments (110 vs. 130 cm) between animals and starting at 130 cm. We selected positions based upon our experience with heifers of similar ages and body sizes.

Heifers were introduced into the experimental pen at 1200 h, approximately 1 h following feed push-up in their home pen. The test heifer remained in the experimental pen for 6 h and was then moved back to the home pen. The test duration was selected based upon data from von Keyserlingk et al. (2011), with the intention that each heifer would use the stall at least once.

Heifer behavior in the experimental area was recorded using one camera (Panasonic WV-BP334 24V, Panasonic, Mississauga, Ontario, Canada) positioned 10 m above the pen surface. Using continuous video, we measured the number of events in which heifers entered the lying stall with only their front 2 hooves and then exited (2 hoof standing), entered with all 4 hooves and then exited without lying down (4 hoof standing), or entered the stall fully and proceeded to lie down (lying in stall). Occasionally heifers turned around in the stall (i.e., backward in stall, facing the alley; either lying or standing), or lay down in the alley. Our predictions were focused on the number of behavioral events, as we were predicting that neck-rail position would primarily affect whether the heifer chose to use the stall, rather than the duration of use.

For descriptive purposes, we also recorded the total time engaged in each of the behavioral outcomes. Similarly, we recorded bedding use and stall cleanliness; these values are not reported below, but all data and code used for analysis are available (see Notes).

The force (N) exerted on the neck rail was measured every 0.1 s throughout the 6-h test period; using these data, the maximum force exerted on the neck rail was identified.

We tested the effect of neck-rail position on the behavioral outcomes (no. of events lying down in the stall, lying down outside of the stall, standing with the front 2 hooves in the stall, standing with all 4 hooves in the stall, inverted in the stall, and the maximum force applied to the instrumented neck rail) using a Mann-Whitney-U test (using the Wilcoxon option within the Npar1way procedure in SAS Studio, v. 3.81, SAS Institute Inc.). We also assessed the Spearman rank correlation (using the Spearman option within the Corr procedure in SAS Studio) between these response variables and the morphometric measures (BW, wither height, hip height, and body length); we saw no evidence of relationships (in all cases *P* > 0.1); these are not reported below but the code is provided in the supplemental materials (see Notes).

How heifers interacted with the stall was affected by neck-rail position (Figure 2). When the neck rail was positioned at 130 versus 110 cm, heifers were more likely to lie down in the stall (Kruskal-Wallis χ^2^ = 4.1, *P* = 0.04). In the work to date most similar to the current study, von Keyserlingk et al. (2011) found no differences in lying time in stalls with and without a neck rail. In studies with older heifers, Sudolar et al. (2017) also found no difference in lying time of heifers housed with access to freestalls with the neck rail positioned at either 150 or 160 cm from the rear curb (based on observations over a 7-d period). The current study, focused on events during the first 6 h after introduction to freestalls, found a median of 3.5 stall entries when the neck rail was positioned at 130 cm from the curb, versus a median of zero entries when the next rail was positioned more restrictively; this result suggests that restrictive neck rails can reduce the likelihood of naïve heifers using the stall for lying, at least initially.

**Figure 2:**
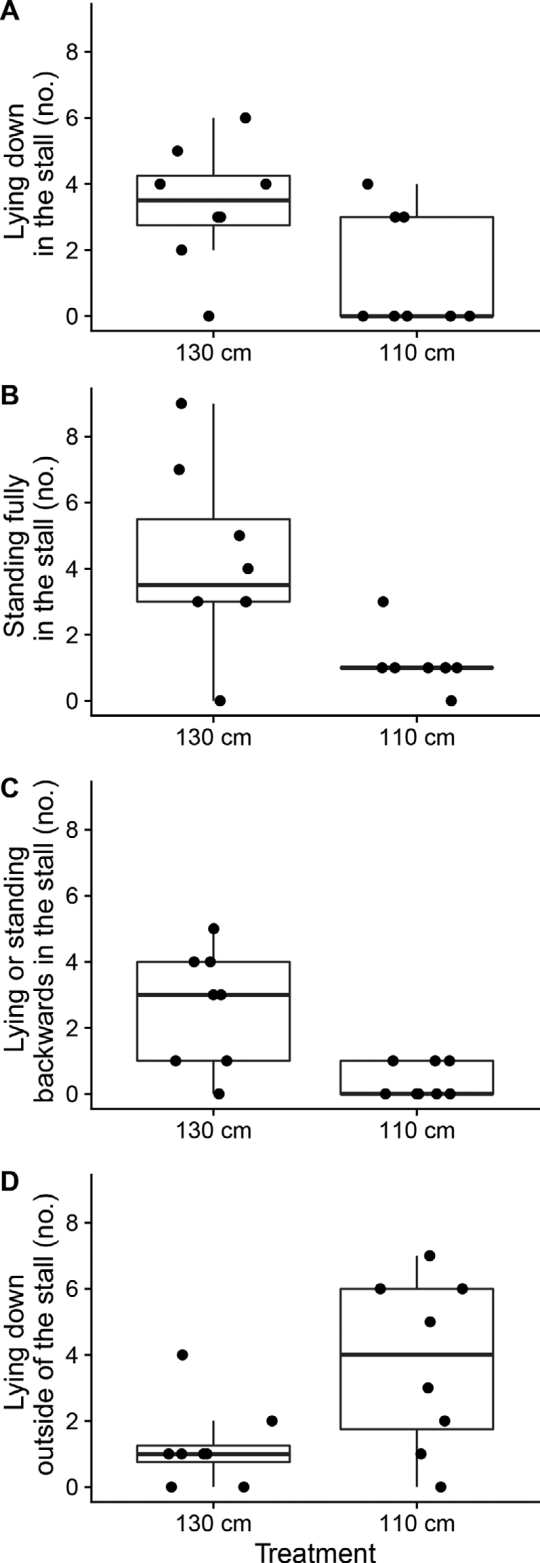
The behavioral responses of heifers during their first 6 h of exposure to a freestall with the neck rail positioned at either 130 or 110 cm from the rear curb (n = 8 heifers per treatment). Responses are shown separately for the number of times heifers (A) lay down inside the freestall, (B) stood fully in the stall facing the neck rail, (C) lay down or stood backward in the stall, and (D) lay down outside of the stall. Box plots show median and 25th and 75th percentiles, whiskers show 10th and 90th percentiles, and individual points show values from individual heifers, jittered to avoid overlap.

We found that heifers were more likely to fully stand inside the stall (i.e., with all 4 hooves on the stall surface) when the neck rail was positioned at 130 cm (χ^2^ = 6.3, *P* = 0.01), but the number of times heifers stood with just 2 hooves on the stall surface was not affected by neck-rail placement (χ^2^ = 0.2, *P* = 0.6). Previous work on how dairy cattle respond to neck-rail placement has shown that less restrictive positioning decreases the likelihood that cattle will stand with just the front 2 hooves in the stall, and increases the ability to stand fully in the stall (Tucker and Weary, 2004; Tucker et al., 2005).

We found that heifers introduced to freestalls with a more restrictive neck rail were more likely to lie down outside of the stall (χ^2^ = 3.9, *P* = 0.05). These results are consistent with previous research on the behavioral responses of naïve heifers to introduction to freestalls (von Keyserlingk et al., 2011; Van Os et al., 2021; both studies measuring responses over several days after introduction), showing that animals spent more time lying down in the alley outside of the stall following introduction to freestalls.

Some heifers were observed standing or lying backward in the stall (i.e., facing the stall entrance), and that this behavior was more likely when heifers were assigned to the 130 cm treatment (χ^2^ = 6.6, *P* = 0.01). Previous work that investigated changes in stall use over time by naïve heifers reported that 85% of the heifers were observed lying forward in the stall (Van Os et al., 2021). These authors also reported variation in how individual heifers adapted to the new environment, and that the risk of this behavior declined over time.

Work with adult dairy cows has shown that providing access to alternative stalls that are less restrictive increases the cow's ability to adopt extended lying positions, such as lying with hind legs extended and with the neck curled back (Beaver et al., 2021). Earlier work by Ruud and Bøe (2011) also showed the benefit of flexible rather than fixed stall features. Stall features that can move when animals contact them can help reduce the force of contact and thus also the risk of any resulting pain or injury.

We found that the naïve heifers exerted considerable forces upon the neck rail. To our knowledge, only one previous study has measured the force with which animals contact the neck rail (Blom et al., 1984), and this study was on adult cattle. This previous study reported that, on average, cattle come into contact with the neck rail 3 times/d (as measured over a 43-d period). Given the lack of data of how often and with what force cattle come into contact with penning, we urge future work to consider such measures. The results of the current study indicate the forces involved can be considerable, and point toward the importance of positioning stall features and designing these in ways that minimize the risk of injury (e.g., by using materials that absorb the force; Gaworski, 2019).

Counter to our prediction, we found that the maximum force applied to the instrumented neck rail tended to be higher when this was positioned at 130 versus 110 cm (χ^2^ = 3.6, *P* = 0.06; Figure 3); the reason for this difference is unclear, but may relate to some heifers standing backward in stalls with the less restrictive neck-rail positioning. We did not match specific force recordings with the heifer's movements, but encourage future work to do so. Some previous work has recorded when cattle come into contact with stall features; for example, Gygax et al. (2005) measured the frequency of impacts against neck rails and stall partitions for stalls of various dimensions. Similarly, Freinberg et al. (2020) assessed instances of contact between cows and stall dividers and neck rails, but only considered contacts with a force greater than 11.3 kg. Hansen et al. (2003) measured force of cows against the feed barrier, helping inform how these barriers should be designed and positioned.

**Figure 3:**
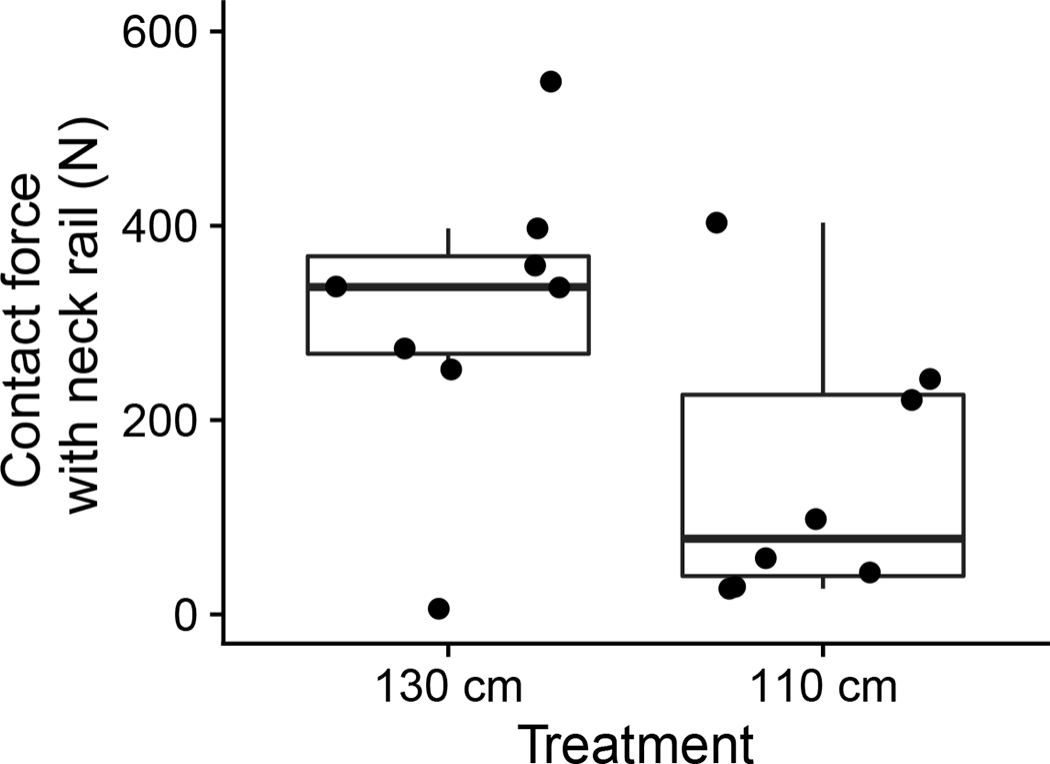
The maximum force applied to the instrumented neck rail by heifers during their first 6 h of exposure to a freestall with the neck rail positioned at either 130 or 110 cm from the rear curb (n = 8 heifers per treatment).

The current study was focused on the responses of heifers during their first 6 h interacting with a freestall. A strength of our method was that this relatively short test period allowed us to test heifers individually, so that responses were unlikely to be affected by the behaviors of other heifers, but the short testing period meant that we were unable to collect meaningful data on stall usage or cleanliness, and testing heifers individually may have affected their behavior. Our 6-h observational period restricts inferences to the acute effects of introduction to the freestall; some behavioral responses likely stabilize after 1 to 3 d (Færevik et al., 2007). Exactly when, where, and how to best record lying behaviors is still debated (Chen et al., 2016; Xiao et al., 2022). We call especially for more longitudinal descriptive research to better understand variation in the ways in which heifers respond to changes in housing over time, potentially considering a wider range or combination of outcomes (Dirksen et al., 2020). Cattle vary greatly in lying behavior (Ito et al., 2009), reinforcing the importance of also understanding these differences in lying behaviors for dairy heifers.

A strength of the current study was the use of automation to directly measure the force applied to the neck rail; our work shows the potential for future, longer-term work instrumenting stalls and including automated methods of tracking individual animals (e.g., Sadrzadeh et al., 2024). A limitation of force measures is that these capture only when the animal's body contacts the instrumented device. In addition to measures of force, future kinematic work is required to better understand the motions of heifers within the stall as they lie down and stand up, along the lines of previous work on mature cattle (e.g., Ceballos et al., 2004). A better understanding of these motions would help understand how animals come into contact with features of the stall, and more generally help inform the design of stalls that are more comfortable.

We measured force as we believed that this would relate to the risks associated with contact with stall hardware. Future work should also measure pressure, and ways of reducing pressure (e.g., by increasing surface area or adding cushioning). Further work is also required to more directly assess harms to the animals, including evidence of contusions and pain.

We conclude that neck-rail position affects the behavior of naïve heifers in the hours following introduction to freestalls; heifers are less likely to use stalls for lying and standing when the rail is positioned more restrictively, and were more likely to spend time lying down outside of the stall. Heifers contacted the neck rail with considerable force, and this force was higher in the stalls with less restrictive neck rails. The position of the neck rail can act as a barrier for young heifers, such that more restrictive neck-rail positions reduce the correct use of the stall. We suggest using a less restrictive neck-rail position when heifers are introduced to freestalls.
